# The importance of parental human papillomavirus vaccine series initiation for reducing sex disparities in human papillomavirus vaccine series initiation among children in the United States

**DOI:** 10.1016/j.pmedr.2025.103160

**Published:** 2025-07-05

**Authors:** Elinita Pollard, Hunter K. Holt, Milkie Vu, Meng-Han Tsai

**Affiliations:** a Georgia Prevention Institute, Augusta University, Augusta, GA, USA; b Center for Health Equity Transformation, University of Kentucky, Lexington, KY, USA; c Department of Family and Community Medicine, University of Illinois College of Medicine, University of Illinois, Chicago, IL, USA; d Department of Preventive Medicine, Feinberg School of Medicine, Northwestern University, Chicago, IL, USA; e Robert H. Lurie Comprehensive Cancer Center, Northwestern University, Chicago, IL, USA; f Cancer Prevention, Control, & Population Health Program, Georgia Cancer Center, Augusta University, Augusta, GA, USA

**Keywords:** Human papillomavirus, Sex disparities, Vaccines, Cancer prevention, HPV-related cancers

## Abstract

**Objective:**

Male children are less likely to initiate the human papillomavirus (HPV) vaccine series than their female counterparts. Furthermore, evidence suggests children with parents who initiated the vaccine series may be more likely to do the same. However, no studies have examined if parents' vaccination status modifies sex differences in HPV vaccine series initiation among children.

**Methods:**

Using the 2022 National Health Interview Survey data, we examined the association between parents' HPV vaccination status (unvaccinated, initiated the vaccine series, unknown) and sex disparities in HPV vaccine series initiation among children using weighted multivariable logistic regression models.

**Results:**

Among 2200 parent-child dyads, less than half of parents (15.6 %) and children (32.6 %) initiated the HPV vaccine series. In adjusted analysis, male children had decreased odds of vaccine series initiation (OR: 0.73, 95 % CI: 0.58, 0.92). Children with parents who initiated the vaccine series had an increased odds of initiating the vaccine series compared to those whose parents were unvaccinated (OR: 2.88, 95 % CI: 2.00, 4.15). When stratified by parent's HPV vaccine series initiation, male children only had a decreased odds of HPV vaccine series initiation among children with unvaccinated parents (OR: 0.69, 95 % CI: 0.54, 0.89).

**Conclusion:**

Male children were only less likely to initiate the HPV vaccine series among children whose parents were unvaccinated against HPV. Thus, healthcare providers should engage both the parent and child in the vaccine recommendation process. Doing so may improve HPV vaccine series initiation for parents who are within the recommended age range and male children.

## Highlights

• Male children were less likely to have initiated the human papillomavirus vaccine (HPV) series overall.
• Children whose parents initiated the HPV vaccine series were more likely to initiate the vaccine series.
• Male children were only less likely to initiate the HPV vaccine series among children whose parents were unvaccinated.

## 1 Introduction

Human papillomavirus (HPV) is the most common sexually transmitted infection in the United States and will infect most sexually active people in their lifetime (Garcia et al., 2024; Jensen et al., 2024; Kreisel et al., 2021). While HPV has been linked to oral, penile, vulvar, anal, vaginal, and cervical cancer (Jensen et al., 2024; National Cancer Institute, 2024), up to 90 % of these cancers can be prevented by HPV vaccine uptake (National Cancer Institute, 2024). The vaccine can be administered as early as the age of nine (Saslow et al., 2020; Meites et al., 2019). The Advisory Committee on Immunization Practices (ACIP) and American Cancer Society (ACS) routinely recommend receiving the HPV vaccine through the age of 26 (Saslow et al., 2020; Meites et al., 2019), with ACIP recommending two doses through the age of 15 and three doses through the age of 26 (Meites et al., 2019). ACIP also recommends shared clinical decision making to identify if individuals through the age of 45 would like to receive the three-dose series (Meites et al., 2019). However, the ACS does not endorse this recommendation (Saslow et al., 2020). Clinical trials surrounding the HPV vaccine focused on cervical cancer and genital diseases among females (Daley et al., 2017; Daley et al., 2016). As such, it was only recommended for females when it was introduced in 2006 (Daley et al., 2017; Daley et al., 2016; Markowitz et al., 2007). Since then, research linking HPV to sex-neutral and male-specific cancers has emerged (Daley et al., 2017; Daley et al., 2016). Thus, HPV vaccination recommendations have been expanded to include males (Villarroel et al., 2024; Centers for Disease Control and Prevention and Advisory Committee on Immunization Practivies, 2011; *MMWR Morb. Mortal Wkly. Rep.*, 2010). This history has likely contributed to extant sex disparities in HPV vaccination coverage with recent data showing male adolescents have lower rates of HPV vaccine series initiation than female adolescents (Villarroel et al., 2024; Pingali et al., 2023).

To understand sex disparities in HPV vaccine series initiation among children, it is important to examine parent-related factors since parents are the primary decision-makers of vaccination in adolescents (Zimet et al., 2021; Silverman et al., 2019). Parental HPV vaccination status may be a critical factor given that prior studies have shown high concordance in parent and child vaccination status for a variety of illnesses. For example, parents who are vaccinated against COVID-19 are more likely to vaccinate their children against COVID-19 (Tu et al., 2023), and parents who have received the flu vaccine are more likely to vaccinate their children against the flu *and* HPV (Robison and Osborn, 2017). However, there is a lack of literature regarding the influence of parental HPV vaccine series initiation on sex disparities in HPV vaccine series initiation among children. Therefore, the objective of this study is to examine if sex disparities in HPV vaccine series initiation among children are modified by parents' vaccination status in the US.

## 2 Methods

### 2.1 Study setting and data source

We used data from the 2022 National Health Interview Survey (NHIS) Sample Adult and Sample Child interviews. NHIS is a cross-sectional interview conducted by the National Center for Health Statistics (NCHS) and collects data on the noninstitutionalized civilians in the United States throughout the year (Centers for Disease Control and Prevention et al., 2022). We used this iteration of NHIS because it is the only one that includes data regarding HPV vaccine series initiation for both adults and children. Survey data is collected for one adult and one child within each household. Adults self-report their data unless they are physically or mentally unable to answer. For children, an adult who is informed about the child's healthcare responds to the interview (Centers for Disease Control and Prevention et al., 2022; Centers for Disease Control and Prevention and National Center for Health Statistics, 2019). NHIS methods and sample selection, including the weighting procedure, are described elsewhere (Centers for Disease Control and Prevention et al., 2022; Centers for Disease Control and Prevention and National Center for Health Statistics, 2019). Because the current study used publicly available de-identified data, it is considered exempt from Institutional Review Board review at Augusta University.

Study sample.

**Fig. 1:**
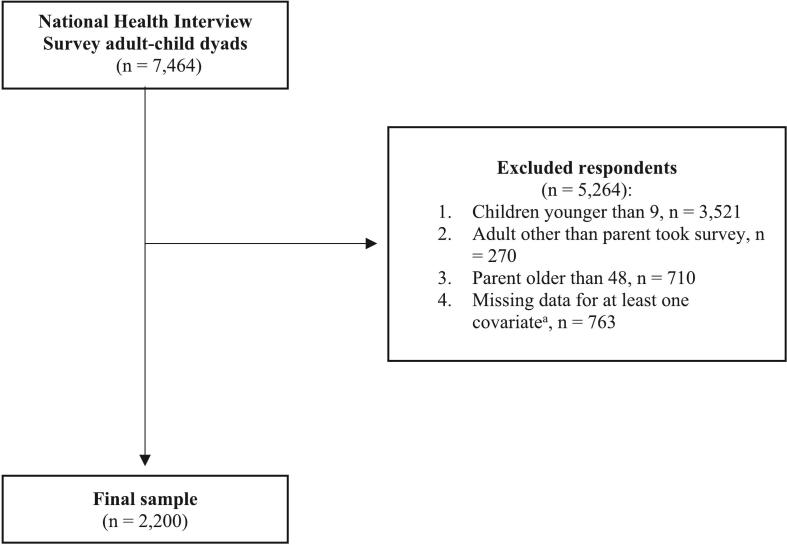
Sample Selection. ^a^Covariates included age, educational attainment, and income to poverty ratio for parent/household characteristics. For child characteristics, we included race/ethnicity and time since the last doctor's visit.

Using household identifiers, we linked 7464 adult-child dyads together. To obtain a study eligible sample, we excluded dyads if the child was younger than 9 years old based on ACIP's guidelines for HPV vaccine series initiation (*n* = 3521) (Meites et al., 2019), an adult other than a parent answered the survey (*n* = 270), the parent was older than 48 (*n* = 710), or there was missing data for at least one covariate (i.e., parent's sex, parent's race, parent's age, parent's educational attainment, the family structure, income to poverty ratio, rural status, parent's insurance status, time since child's last doctor visit, and the child's age, *n* = 763). We only included parents in the analyses as they are typically in charge of making medical decisions for their own child (Silverman et al., 2019). Therefore, including other adult-child dyads could skew responses as other adults may not be as involved in the child's medical decision making process. Further, we only included parents who were 48 and younger because ACIP released updated recommendations that reported the vaccine was approved for people through the age of 45 in 2019. In 2022, the oldest newly approved person would be 48. Thus, we excluded parents older than 48 because they were never young enough to receive the vaccine based on ACIP guidelines (Meites et al., 2019). As a result, our study sample included 2200 parent-child dyads **(**Fig. 1**)**.

### 2.2 Variables of interest

Our primary outcome of interest was parent-reported child HPV vaccine series initiation based on an NHIS item: “HPV is the Human Papillomavirus. Has [child's name] ever received an HPV shot or vaccine?” Children whose parents responded “yes” were considered to have initiated the HPV vaccine series (termed as “reported child vaccine series initiation”). Children whose parents responded “no,” refused to answer, did not know their child's vaccination status, were coded as “not ascertained,” or missing were categorized as not having reported vaccine series initiation (termed as “no reported child vaccine series initiation”). The child's sex and the parent's HPV vaccination status were our main exposures of interest. The child's sex was classified as “male” or “female.” Parents' vaccination status was determined from a single NHIS question “HPV is the Human Papillomavirus. Have you ever received an HPV shot or vaccine?” Parents who responded “yes” were categorized as having initiated the vaccine series (termed as initiated) whereas those who responded “no” were classified as “unvaccinated.” Parents whose vaccination status could not be ascertained (e.g., missing, refused, not ascertained, did not know) were classified as “unknown.” We included unknown as a category because it allows to examine whether an unknown vaccination status impacted child vaccination status.

For covariates of interest, we included information regarding parental characteristics, household characteristics, parent-child access to care factors, and the child's age. Parental characteristics included parents' sex (male, female), race (Hispanic, non-Hispanic Black, non-Hispanic Other, non-Hispanic White), age (18–39, 40–48 years), educational attainment (less than a bachelor's, bachelor's or more). Household characteristics included the family structure (single parent, more than one adult, or unknown), income to poverty ratio (0–1.99, 2,00–3.99, or 4.00+) based on the Census Bureau's poverty thresholds from the previous calendar year, and urban-rural classification based on the NCHS' classification scheme (nonmetropolitan, medium and small metropolitan, large fringe metro, or large central metro). We included parents' insurance status (uninsured, insured) and the time since the child's last doctor visit (within the past year, ≥ 1 year ago) as parent-child access to care factors. Finally, we considered the child's age (9–12, 13–14, 15–17 years).

### 2.3 Statistical analysis

We conducted weighted analyses in line with NHIS guidelines (Centers for Disease Control and Prevention et al., 2022). We used a crosstabulation of frequencies and weighted percentages to describe differences in the child's sex, parent vaccination status, parental characteristics, household characteristics, parent-child access to care factors, and the child's age for the entire sample with child vaccination status by using Rao-Scott chi square tests. We also examined bivariate associations in vaccine series initiation based on the child's sex, parental characteristics, household characteristics, parent-child access to care factors, and the child's age stratified by parent HPV vaccination status by using Rao-Scott chi square tests. Next, we ran sequential multivariable logistic regression models to assess the association between child's sex, parent vaccination status, and child vaccination status. The crude model included child's sex, parent vaccination status only; model 1 was further adjusted for parental characteristics and the child's age; model 2 was further adjusted for household characteristics; model 3 was further adjusted for parent-child access to care factors. Then, we conducted a stratified analysis to examine sex disparities in vaccine series initiation among children within each level of parental vaccination status. Models for stratified analyses were adjusted for parental characteristics, the child's age, household characteristics, and parent-child access to care factors. We conducted a false discovery rate analysis to adjust for the potential occurrence of a Type I error. This adjustment provides a less conservative approach by controlling the expected proportion of false positives among the rejected hypotheses. Finally, we conducted a supplementary multivariable logistic regression with parents who were unvaccinated and had an unknown vaccination status combined into one group. We did so to create consistency in how parents' and child's HPV vaccination status was operationalized. The purpose of this additional analysis enables to explain the result differences when using unvaccinated and unknown vaccination as separate groups in main analysis. Results are reported as odds ratios (ORs) and their corresponding 95 % confidence intervals (CIs). We considered differences to be significant when *p* < 0.05 using two-sided probability tests. SAS Version 9.4, SAS Institute Inc., Cary, North Carolina was used for all analyses.

## 3 Results

### 3.1 Sample characteristics

Most parents (77.5 %) were unvaccinated and did not report vaccine series initiation for their children (67.4 %). Around half of the sample reported data for a male child (53.2 %). Parents were most frequently female (57.4 %), non-Hispanic White (49.3 %), 40–48-years-old (50.8 %), and had less than a bachelor's degree (54.5 %). Most households had more than one adult in the home (75.4 %). Around a third of our sample had an income-to-poverty ratio of 0 to 1.99 (37.2 %) and lived in large central metropolitan areas (30.7 %). Most parents were insured (84.0 %) and had at least a doctor's visit for a child in the past year (90.6 %). Around half of the children were between 9 and 12 years old (48.7 %). There were significant differences in child vaccine series initiation by parental vaccination status and child's sex (both *p* < 0.01). While unvaccinated parents only made up 77.5 % of the sample, they only comprised 68.7 % of the parents who reported vaccine series initiation for their children. In contrast, parents who reported vaccine series initiation made up 15.6 % of the sample, yet they comprised 23.7 % of those who reported vaccine series initiation for their children. Finally, parents with an unknown vaccination status made up 6.9 % of the sample but 7.6 % of those who reported child vaccine series initiation. While male children made up a larger proportion of the sample (53.2 % vs 46.8 %), a smaller proportion of them had initiated the vaccine series compared to female children (47.5 % vs 52.5 %; Table 1).

**Table 1:** Parents' human papillomavirus vaccination status, parents' demographics, children's characteristics, and household factors by children's human papillomavirus vaccine series initiation in the United States, 2022.

	Total (*n* = 2200)	No reported child vaccine initiation (*n* = 1447, 67.4 %)	Reported child vaccine initiation (*n* = 753, 32.6 %)	*P*-Value
**N(%)**^a^				
**Parent vaccination status**				<0.01
Unvaccinated	1720(77.5 %)	1181(81.8 %)	539(68.7 %)	
Initiated	335(15.6 %)	172(11.7 %)	163(23.7 %)	
Unknown	145(6.9 %)	94(6.5 %)	51(7.6 %)	
**Child's sex**				<0.01
Male	1155(53.2 %)	796(55.9 %)	359(47.5 %)	
Female	1045(46.8 %)	651(44.1 %)	394(52.5 %)	
*Parental characteristics*				
**Sex**				0.24
Male	857(42.6 %)	583(43.6 %)	274(40.5 %)	
Female	1343(57.4 %)	864(56.4 %)	479(59.5 %)	
**Race**				0.45
Hispanic	559(30.1 %)	388(31.4 %)	171(27.5 %)	
Non-Hispanic Black	219(9.7 %)	140(9.4 %)	79(10.4 %)	
Non-Hispanic Other	254(10.9 %)	170(11.0 %)	84(10.7 %)	
Non-Hispanic White	1168(49.3 %)	749(48.3 %)	419(51.3 %)	
**Age**				
18–39	973(49.2 %)	682(51.5 %)	291(44.7 %)	0.02
40–48	1227(50.8 %)	765(48.5 %)	462(55.3 %)	
**Education**				0.21
Less than a bachelor's	1133(54.5 %)	768(55.6 %)	365(52.2 %)	
Bachelor's or more	1067(45.5 %)	679(44.4 %)	388(47.8 %)	
*Household Characteristics*				
**Family structure**				0.11
Single parent	552(15.3 %)	362(15.0 %)	190(16.0 %)	
More than one adult	1464(75.4 %)	973(76.7 %)	491(72.6 %)	
Unknown	184(9.3 %)	112(8.3 %)	72(11.4 %)	
**Income to poverty ratio**				<0.01
0–1.99	710(37.2 %)	502(39.4 %)	208(32.2 %)	
2.00–3.99	637(29.2 %)	433(30.2 %)	204(27.2 %)	
4.00+	853(33.6 %)	512(30.2 %)	341(40.6 %)	
*Rurality*				
Nonmetropolitan	305(12.4 %)	214(13.2 %)	91(10.8 %)	0.45
Medium and small metro	666(27.6 %)	435(26.6 %)	231(29.8 %)	
Large fringe metro	605(29.2 %)	382(29.3 %)	223(29.1 %)	
Large central metro	624(30.7 %)	416(30.9 %)	208(30.4 %)	
*Parent-Child Access to Care*				
**Parent's insurance status**				0.07
Uninsured	290(16.0 %)	205(17.3 %)	85(13.2 %)	
Insured	1910(84.0 %)	1242(82.7 %)	668(86.8 %)	
**Child's last doctor visit**				<0.01
Within the past year	2014(90.6 %)	1298(88.9 %)	716(94.3 %)	
≥1 year ago	186(9.4 %)	149(11.1 %)	37(5.7 %)	
**Child's age**				<0.01
9–12	1038(48.7 %)	853(59.8 %)	185(25.9 %)	
13–14	472(21.4 %)	252(17.8 %)	220(28.8 %)	
15–17	690(29.9 %)	342(22.4 %)	348(45.3 %)	

a Data shown as unweighted frequencies and weighted percentages. Column percentages were used.

Table 2 shows differences in child vaccine series initiation based on parental vaccination status. Data is only shown for children who had reported HPV vaccine series initiation. Among children of unvaccinated parents, 28.9 % initiated the vaccine series. In contrast, among children of vaccinated parents, 49.5 % initiated the vaccine series. Among children of parents with unknown vaccination status, 36.1 % initiated the vaccine series. Among children with unvaccinated parents, vaccine series initiation was significantly higher for females (33.1 %) than males (25.0 %; *p* = 0.001). A similar pattern was observed among children of initiated parents: 58.4 % of females initiated the vaccine series compared to 42.8 % of males (*p* = 0.02). However, sex disparities were not significant among children whose parents' vaccination status was unknown (*p* = 0.17).

**Table 2:** Frequencies and weighted percentages of HPV vaccine series initiation among children based on child's sex, parent characteristics, household characteristics, and child characteristics by parent vaccination status in the United States, 2022.^a^

	Unvaccinated Parents (*n* = 539, 28.9 %)		Initiated Parents (*n* = 163, 49.5 %)		Unknown Parents (*n* = 51, 36.1 %)	
	n(%)	P-value	n(%)	P-value	n(%)	P-value
**Child's sex**		<0.01		0.022		0.17
Male	254(25.0)		80(42.8)		25(41.7)	
Female	285(33.1)		83(58.4)		26(29.8)	
*Parental Characteristics*						
**Sex**		0.39		0.18		0.83
Male	212(27.7)		39(57.1)		23(35.1)	
Female	327(29.8)		124(46.9)		28(37.0)	
**Race**		0.05		0.45		0.94
Hispanic	112(24.1)		43(50.2)		16(38.5)	
Non-Hispanic Black	51(29.8)		22(58.0)		6(29.3)	
Non-Hispanic Other	49(24.7)		26(55.7)		9(34.7)	
Non-Hispanic White	327(32.1)		72(44.4)		20(36.2)	
**Age**		<0.01		0.01		0.78
18–39	159(23.4)		109(45.0)		23(34.8)	
40–48	380(33.0)		54(64.3)		28(37.8)	
**Education**		0.08		0.09		0.46
Less than a bachelor's	257(26.8)		84(49.2)		23(39.2)	
Bachelor's or more	282(31.4)		79(50.0)		27(33.1)	
*Household Characteristics*						
**Family structure**		0.02		0.82		0.66
Single parent	130(30.8)		49(46.0)		11(26.0)	
More than one adult	361(27.7)		94(50.7)		36(37.6)	
Unknown	48(36.6)		20(49.9)		4(38.8)	
**Income to poverty ratio**		<0.01		0.72		0.47
0–1.99	144(23.9)		51(48.3)		13(32.9)	
2.00–3.99	131(25.0)		54(47.2)		19(44.7)	
4.00+	264(37.4)		58(53.3)		19(32.0)	
**Rurality**		0.35		0.93		0.83
Nonmetropolitan	72(25.1)		15(47.6)		4(31.3)	
Medium and small metro	170(32.2)		47(52.9)		14(36.9)	
Large fringe metro	157(29.0)		56(47.3)		10(28.6)	
Large central metro	140(27.2)		45(50.2)		23(39.7)	
*Parent-Child Access to Care*						
**Parent's Insurance Status**		0.07		0.18		0.25
Uninsured	66(23.3 %)		16(64.9 %)		3(20.8)	
Insured	473(30.1 %)		147(47.9 %)		48(37.6)	
**Child's Last Doctor Visit**		<0.01		0.98		0.18
Within the past year	513(30.2 %)		154(49.5 %)		49(37.2)	
≥1 year ago	26(17.0 %)		9(49.3 %)		2(17.1)	
**Child's age**		<0.01		<0.01		0.04
9–12	136(15.1 %)		36(27.3 %)		13(20.9)	
13–14	172(40.7 %)		35(64.3 %)		13(44.6)	
15–17	231(43.5 %)		92(71.9 %)		25(52.0)	

*P*-values are reflective of comparisons of children with parent-reported vaccine series initiation to children without parent-reported HPV vaccine series initiation.

a Data shown as unweighted frequencies and weighted percentages. Row percentages were used. Data not shown for children without reported HPV vaccine series initiation.

### 3.2 Association between the child's sex, parent vaccination status, and child vaccination status

As shown in Table 3, results from the unadjusted model were similar to results from the full model. In the fully-adjusted model (model 3), male children had a decreased odds of vaccine series initiation compared to female children (OR: 0.73, 95 % CI: 0.58, 0.92). Moreover, parents who initiated the HPV vaccine series had a 2.88 increased odds of having a child who was vaccinated against HPV compared to parents who were unvaccinated (OR: 2.88, 95 % CI: 2.00, 4.15). In our supplementary analysis, we observed similar results; male children and children with unvaccinated/unknown parents had a decreased odds of HPV vaccine series initiation **(Supplementary Table 1)**.

**Table 3:** Association between children's sex and parents' human papillomavirus vaccination status on children's human papillomavirus vaccination status in the United States, 2022.

	n/N(%)^a^	Unadjusted Model^b^	Fully Adjusted Model^b^
*Sex of child*			
Female	394/1045(36.5 %)	1.00	1.00
Male	359/1155(29.1 %)	0.69(0.56, 0.85)	0.73(0.58, 0.92)
*Parent vaccination status*			
Unvaccinated	539/1720(28.9 %)	1.00	1.00
Initiated	163/335(49.5 %)	2.48(1.85, 3.31)	2.88(2.00, 4.15)
Unknown	51/145(36.1 %)	1.40(0.84, 2.34)	1.41(0.79, 2.53)

a Data shown as unweighted frequencies and weighted percentages.

b Weighted logistic regressions were used in all models. The unadjusted model included the sex of the child and parental vaccination status. The fully adjusted model was adjusted for parental characteristics, children's ages, household characteristics, and parent-child access to care variables. Data shown as odds ratios and 95 % confidence intervals.

Among children with unvaccinated parents, male children had a 0.69 decreased odds of vaccine series initiation compared to female children (OR: 0.69, 95 % CI: 0.54, 0.89). However, sex disparities were not observed among with children whose parents initiated the vaccine series (OR: 0.65, 95 % CI: 0.35, 1.21) or had an unknown vaccination status (OR: 1.47, 95 % CI: 0.57, 3.74; Table 4). Our false discovery rate analysis indicated the association between the child's sex and HPV vaccine series initiation was only significant for those with unvaccinated parents (*p* = 0.02). These findings align with our original analysis, suggesting a reduced likelihood of a Type I error. In our supplementary analysis, male children only had a lower odds of vaccine series initiation among those with parents who were unvaccinated/unknown **(Supplementary Table 2)**.

**Table 4:** Association between parents' vaccination status and vaccine series initiation among children stratified by sex in the United States, 2022.

	Unvaccinated Parents		Initiated Parents		Unknown Parents	
	n/N(%)	OR(95 % CI)	N(%)	OR(95 % CI)	N(%)	OR(95 % CI)
*Sex of child*						
Female	285/826(33.1 %)	1.00	83/146(58.4 %)	1.00	26/73(29.8 %)	1.00
Male	254/894(25.0 %)	0.69(0.54, 0.89)	80/189(42.7 %)	0.65(0.35, 1.21)	25/72(41.7 %)	1.47(0.57, 3.74)

Abbreviations: CI: confidence interval; OR: odds ratio.

Weighted frequencies and percentages are shown for children with reported human papilloma virus vaccine series initiation.

Weighted logistic regressions were used in all models. All models were adjusted for parental characteristics, children's ages, household characteristics, and parent-child access to care.

## 4 Discussion

In the current study, we found male children had decreased odds of HPV vaccine series initiation compared to female children overall. In a recent report, Villarroel and colleagues reported similar results: 34.6 % of 9- to 17-year male children had reported vaccine series initiation compared to 42.9 % of old female children in the same age range (Villarroel et al., 2024). Sex disparities in HPV vaccination series initiation may be due in part to what Daley and colleagues refer to as the “feminization” of HPV (Daley et al., 2017; Daley et al., 2016). Historically, HPV vaccine research was centered around cervical cancer (Daley et al., 2016). Thus, the vaccine was initially only recommended for females when it was introduced in 2006 (Daley et al., 2017; Daley et al., 2016; Markowitz et al., 2007). The vaccine was not approved for males until 2009 and was not routinely recommended to them until 2011 (Daley et al., 2017; Centers for Disease Control and Prevention and Advisory Committee on Immunization Practivies, 2011; *MMWR Morb. Mortal Wkly. Rep.*, 2010). The feminization of HPV may partially explain why national data has shown adult males are less likely to be aware of HPV (Adjei Boakye et al., 2017; Osazuwa-Peters et al., 2017), the HPV vaccine (Adjei Boakye et al., 2017; Osazuwa-Peters et al., 2017), and to be vaccinated (Rincon et al., 2024; McElfish et al., 2021). This pattern is concerning, especially given that in recent years, rates of oropharyngeal cancers in males have exceeded cervical cancer rates (Liao et al., 2022; Lechner et al., 2022; Roman and Aragones, 2021; Centers for Disease Control and Prevention, 2025). Given sex disparities in HPV vaccine series initiation exist among adults and its decreased efficaciousness with age (Jensen et al., 2024; Rincon et al., 2024; McElfish et al., 2021; Burger et al., 2017; Ellingson et al., 2023), it is critical to direct efforts to ameliorate sex disparities in vaccine series initiation among children. Doing so has the potential to mitigate the burden of HPV cancers that are currently becoming more common.

Our study provides novel insight into the concordance between parent and child HPV vaccine series initiation status. Previous studies have shown parent/child vaccine concordance for other vaccines such as the flu and COVID-19 vaccines (Tu et al., 2023; Robison and Osborn, 2017). We observed a similar pattern in our study; children with parents who initiated the vaccine series also had increased odds of vaccine series initiation. Past research has also shown that attitudes, knowledge, and beliefs about the HPV vaccine significantly influence vaccine intentions and series initiation in adults and children (Mansfield et al., 2021; O'Leary et al., 2018; Lacombe-Duncan et al., 2018; Newman et al., 2018; Thompson et al., 2021; Mansfield et al., 2018; Moss et al., 2015; Beavis et al., 2024). Taken together (Mansfield et al., 2021; O'Leary et al., 2018; Lacombe-Duncan et al., 2018; Newman et al., 2018; Thompson et al., 2021; Mansfield et al., 2018; Moss et al., 2015; Beavis et al., 2024), our findings suggest parents and children who initiated the vaccine series may have higher levels of HPV-related knowledge and more positive attitudes regarding the vaccine. However, we were unbale to examine the impact of knowledge and attitudes regarding the vaccine due to the unavailability of relevant data. Thus, further research in this area is warranted.

Though other studies have examined parent/child concordance for other vaccines and sex disparities in HPV vaccine series initiation have been identified (Villarroel et al., 2024; Tu et al., 2023; Robison and Osborn, 2017), no studies have examined the potential impact of parental HPV vaccine series initiation on sex disparities in HPV vaccine series initiation among children. We found Sex disparities in vaccine series initiation were only present among male children with unvaccinated parents in adjusted analyses. This raises the concern that male children with unvaccinated parents may have consistently faced disproportionate barriers to HPV vaccine series initiation (e.g., lack of healthcare provider recommendation) (Kong et al., 2021; Lindley et al., 2016; Finney Rutten et al., 2017). Therefore, future researchers should examine sex-specific barriers to vaccine series initiation, and efforts to implement proven facilitators of vaccine series initiation such as HPV-related education and healthcare provider recommendations should be delivered in an equitable manner (Finney Rutten et al., 2017; Oh et al., 2021; Gilkey et al., 2016; Dixon et al., 2019).

### 4.1 Implications

High-quality recommendations from healthcare providers have been associated with higher rates of HPV vaccine series initiation and completion (Finney Rutten et al., 2017; Oh et al., 2021; Gilkey et al., 2016). Gilkey and colleagues' findings suggest healthcare providers should highly endorse the vaccine, highlight its potential for cancer prevention, and recommend same-day vaccination (Gilkey et al., 2016). Despite the influence of provider recommendations on vaccine initation (Finney Rutten et al., 2017; Oh et al., 2021; Gilkey et al., 2016), previous studies have consistently shown parents of male children were less likely to have a healthcare provider recommend the vaccine for their child (Kong et al., 2021; Lindley et al., 2016; Finney Rutten et al., 2017). Therefore, healthcare providers should be more cognizant of providing high-quality recommendations for the vaccine for male children. Additionally, our findings imply incorporating family members, specifically parents, in the vaccine recommendation process has the potential to ameliorate sex disparities in HPV vaccine initiation among children. Educational interventions are a viable way to do so and have the potential to reach children who have limited access to care.

Prior educational interventions have been successfully implemented in settings that are outside of the healthcare system, including but not limited to, virtual settings and schools (Dike et al., 2023; Panozzo et al., 2020; Yoost et al., 2017). Previous studies have shown providing parents, children, and healthcare personnel with education about HPV has been shown to foster more positive attitudes and improve knowledge regarding the vaccine for parents and children (Dike et al., 2023; Ortiz et al., 2018; Reiter et al., 2011); As such, educational interventions have been linked to intention to vaccinate and vaccine series initiation for children (Dixon et al., 2019; Panozzo et al., 2020; Yoost et al., 2017). Similar to our implications for provider recommendations, a recent scoping review concluded HPV-related interventions should include the parent and child (Walker et al., 2024). Future educational interventions may inform parents who are within the recommended age range that they can receive the vaccine as well and encourage them to talk to their healthcare provider about it. Our results indicate this may indirectly boost rates of vaccine series initiation and combat sex disparities among children. Additionally, more interventions targeting parent-son dyads are needed. Efforts such as these may improve persistent sex disparities in vaccine series initiation among children.

### 4.2 Strengths and limitations

Our study was the first to examine the association between parental HPV vaccine series initiation and child HPV vaccine series initiation while also considering the child's sex. Since we stratified our analyses by the parents' vaccination status, our findings can inform primary care initiatives and education programs aimed at improving child vaccination through enhancing parent vaccine initiation. Moreover, the use of a nationally weighted sample allows our results to be generalized to the entire United States rather than a specific region. Finally, rather than only adjusting for parental factors, we adjusted for factors associated with parents, children, and the household. By adjusting for multifaceted factors, we were able to capture a more accurate depiction of the relationships we examined.

Nevertheless, we acknowledge a few limitations. Due to the cross-sectional nature of the current study, temporal relationships could not be established between parent vaccination status and child vaccine series initiation. Future researchers may be interested in examining whether the parent or the child initiated the vaccine series first. Understanding this dynamic is crucial for developing targeted educational interventions to improve vaccine initiation among both parents and children. Moreover, self-reported data may lead to under or overreporting of HPV vaccine initiation among parents and children due to recall bias and social desirability bias. Given that there was only one year of data available regarding HPV vaccine series initiation for adults and children, we are unable to determine if pattern in this study have persisted over time, particularly before and after the updated recommendations for male children. Due to the large number of children who initiated the HPV vaccine series, the use of odds ratios rather than prevalence ratios may may lead to an overestimation of the associations examined; it is important to note that odds ratios measure associations but do not directly represent prevalence ratios. Despite this limitation, we employed traditional logistic regression to enhance the comparability and interpretability of our findings. Finally, we did not include how many doses the parent or child received because this information is not available in NHIS. However, it is important to note one dose of the vaccine has been shown to be efficacious (Stanley et al., 2024). Still, those who received the recommended number of doses may differ from parents who only initiated the vaccine series in terms of knowledge and attitudes regarding the vaccine. Factors such as these may influence parents' decision to vaccinate their children and should be further examined. More importantly, healthcare resources, and perceptions surrounding HPV, and the vaccine play important roles in children vaccine initiation (Mansfield et al., 2021; Lacombe-Duncan et al., 2018; Newman et al., 2018; Moss et al., 2015); thus, future research examining these factors may reduce sex disparities.

## 5 Conclusion

Male children had lower likelihood of having vaccine series initiation overall. Contrarily children whose parent initiated the vaccine series had a greater likelihood of vaccine initiation. Sex disparities in HPV vaccine series initiation were only observed among children whose parents were unvaccinated. Therefore, healthcare providers should aim to be conscious of engaging both the parent and child in the vaccine recommendation process and recommending the vaccine to male children. Researchers should also aim to employ more educational interventions that target parent-son dyads. Efforts such as these may improve vaccine series initiation among parents and children as well as combat sex disparities in vaccine series initiation among children.

## Disclosure of Ethical Compliance

The current study is considered exempt from Institutional Review Board (IRB) review at Augusta University due to the use of publicly available de-identified data.

## Funding Credits and Disclosure of Potential and Real Conflicts of Interest

No funding is directed to Drs. Holt or Vu for this work. Dr. Holt was supported by the University of Illinois at Chicago (UIC)‘s Building Interdisciplinary Research Careers in Women's Health (BIRCWH) grant (K12HD101373) from the National Institutes of Health (NIH) Office of Research on Women's Health (ORWH). Dr. Vu was supported by the 10.13039/100000054National Cancer Institute (1K01CA296781-01).Dr. Tsai was supported at least in part through the Georgia Cancer Center Paceline funding mechanism at Augusta University (MCGFD01071) and National Cancer Institute (NCI, R21CA301113). The authors have no conflicts of interest to disclose.

## CRediT authorship contribution statement

**Elinita Pollard:** Writing – review & editing, Writing – original draft, Methodology, Investigation, Formal analysis, Conceptualization. **Hunter K. Holt:** Writing – review & editing. **Milkie Vu:** Writing – review & editing. **Meng-Han Tsai:** Writing – review & editing, Supervision, Methodology, Conceptualization.

## Declaration of competing interest

The authors declare that they have no known competing financial interests or personal relationships that could have appeared to influence the work reported in this paper.

## Data Availability

Publicly available data, so everyone has access to.

## Appendix A Supplementary data

Supplementary Table 1. Association between children’s sex and parents’ human papillomavirus vaccination status on children’s HPV vaccination status. Supplementary Table 2. Association between parents’ vaccination status and vaccine uptake among children stratified by sex Image 1
