# Supplementary material for: The importance of parental human papillomavirus vaccine series initiation for reducing sex disparities in human papillomavirus vaccine series initiation among children in the United States
Source: Prev Med Rep. 2025 Jul 5;56:103160. doi: 10.1016/j.pmedr.2025.103160 (PMC12275476; doi:10.1016/j.pmedr.2025.103160)
Supplement: Supplementary file 1 — Supplementary Table 1. Association between children’s sex and parents’ human papillomavirus vaccination status on children’s HPV vaccination status. Supplementary Table 2. Association between parents’ vaccination status and vaccine uptake among children stratified by sex [file mmc1.docx]

**Supplementary Table 1. Association between children’s sex and parents’ human papillomavirus vaccination status on children’s HPV vaccination status**

|  | **Crude Model** | **Model 1^a^** | **Model 2^a^** | **Model 3^a^** |
| --- | --- | --- | --- | --- |
| **OR(95% CI)** | | | | |
| **Child’s sex** |  |  |  |  |
| Female | Reference | Reference | Reference | Reference |
| Male | **0.69(0.56-0.86)** | **0.72(0.57-0.91)** | **0.72(0.57-0.91)** | **0.73(0.58-0.92)** |
| **Parent vaccination status** |  |  |  |  |
| Unvaccinated or  Unknown | Reference | Reference | Reference | Reference |
| Initiated | **2.45(1.80-3.21)** | **2.98(2.08-4.26)** | **2.89(2.01-4.15)** | **2.77(1.91-4.01)** |

Abbreviations: CI: confidence interval; OR: odds ratio

^a^Weighted logistic regressions were used in all models. Model 1 was adjusted for parental characteristics and the children’s ages. Model 2 was adjusted for further adjusted for household characteristics. Model 3 was further adjusted for parent-child access to care variables.

**Supplementary Table 2.** **Association between parents’ vaccination status and vaccine uptake among children stratified by sex**

|  | **Unvaccinated/Unknown Parents** | | **Vaccinated Parents** | |
| --- | --- | --- | --- | --- |
|  | **N(%)** | **OR(95% CI)** | **N(%)** | **OR(95% CI)** |
| **Child’s sex** |  |  |  |  |
| Female | 311(32.8%) | Reference | 83(58.4%) | Reference |
| Male | 279(26.4%) | **0.76(0.59-0.97)** | 80(42.8%) | 0.65(0.35-1.21) |

Abbreviations: CI: confidence interval; OR: odds ratio

Weighted frequencies and percentages are shown for children with reported human papilloma virus vaccine uptake.

Weighted logistic regressions were used in all models. All models were adjusted for parental characteristics, children’s ages, household characteristics, and parent-child access to care.
